# Screening for natural products that affect Wnt signaling activity

**DOI:** 10.1007/s11418-019-01320-9

**Published:** 2019-05-30

**Authors:** Masami Ishibashi

**Affiliations:** Graduate School of Pharmaceutical Sciences, Chiba University, 1-8-1 Inohana, Chuo-ku, Chiba, 260-8675 Japan

**Keywords:** Natural products, Screening, Wnt, Plants

## Abstract

Wnt signaling has been implicated in numerous aspects of development, cell biology, and physiology. When aberrantly activated, Wnt signaling can also lead to the formation of tumors. Thus, Wnt signaling is an attractive target for cancer therapy. Based on our screening program targeting Wnt signaling activity using a cell-based luciferase screening system assessing TCF/β-catenin transcriptional activity, we isolated a series of terpenoids and heterocyclic aromatic compounds that affect the Wnt signaling pathway at different points. Here, we describe our recent results in screening for natural products that inhibit or activate Wnt signaling.

## Introduction

In our efforts to identify bioactive natural products [1], we recently examined screening programs to isolate compounds targeting biological pathways such as Wnt, Hedgehog, Notch [2], and TRAIL [3] from various natural resources, including microorganisms (Myxomycetes [4] and Actinomycetes [5]) and plants collected from South Asian countries [6, 7]. We also used a synthetic-compound library with natural product-based structures that was constructed by our group for screening studies [8–10]. Here, we describe our recent studies screening for natural products that affect the Wnt signaling pathway [11].

**Fig. 1:**
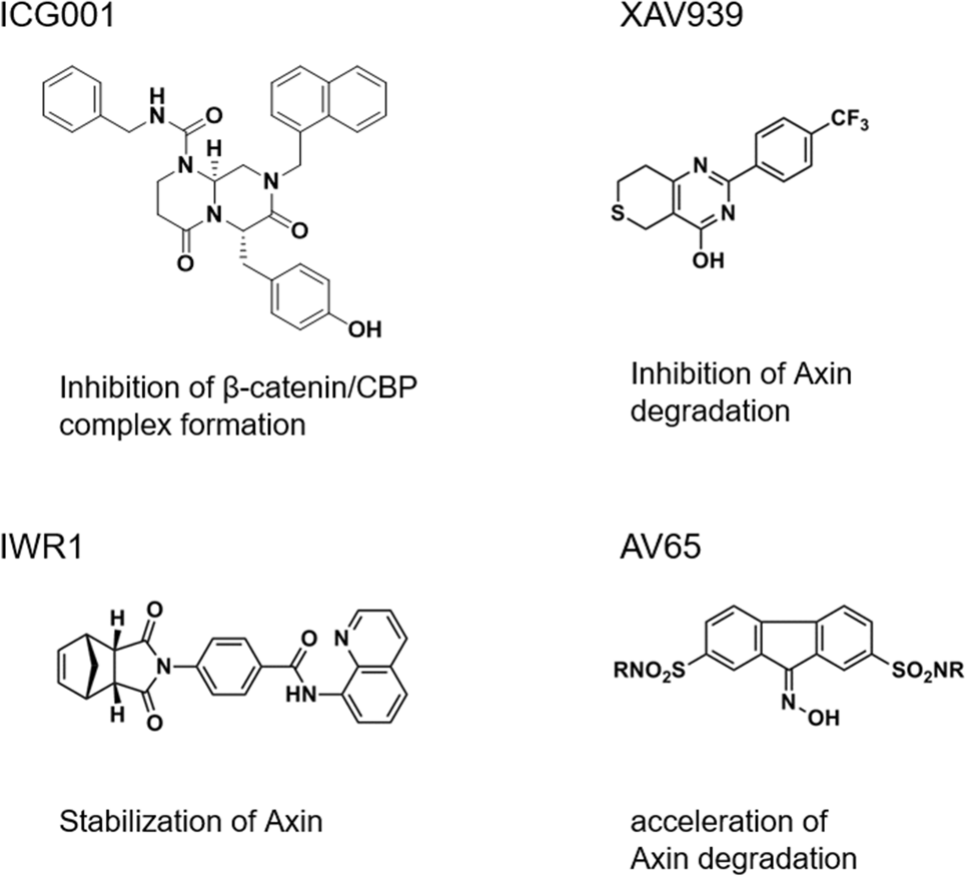
Small-molecule Wnt signaling inhibitors

Wnt signaling is conserved among a variety of species and has been implicated as playing a role in numerous aspects of development, cell biology, and physiology. Wnt signaling has been implicated not only in tumorigenesis, but also diabetes, schizophrenia, and other disorders. Thus, the Wnt signaling pathway is an attractive therapeutic target, and there is significant interest in identifying Wnt signaling inhibitors from libraries of small-molecule natural products. Figure 1 illustrates previously reported Wnt signaling inhibitors, most of which are synthetic compounds that affect different steps in the Wnt signaling pathway [12, 13]. We have screened a variety of natural product extracts [14–16] and synthetic compound libraries [17–19] to identify compounds that target Wnt signaling.

## Screening method

A cell-based reporter luciferase assay system that monitors inhibition of TCF/β-catenin transcriptional activity (TOP activity) was used to screen for compounds that target Wnt signaling. The TCF/β-catenin complex is a downstream transcriptional factor in the Wnt pathway [14]. TOP activity was monitored using STF/293 cells, which are 293 human embryonic kidney cells stably transfected with SuperTOP-flash [luciferase reporter plasmid containing eight copies of the TCF-binding site (CCTTTGATC)]. The cells were kindly provided by Prof. J. Nathans (John Hopkins University). The reporter luciferase is activated by addition of lithium chloride, an inhibitor of GSK3β (glycogen synthase kinase 3β). The Wnt signaling inhibition activity of the sample is assessed by the decrease in luciferase activity. SuperFOP-flash, which has a mutant TCF binding site (CCTTTGGCC), was provided by Prof R. T. Moon (University of Washington) and used to evaluate and exclude non-selective inhibition of Wnt signaling based on monitoring of luciferase activity (FOP activity). Using this assay system, hundreds of plant extracts were examined by our group, resulting in the selection of several hit samples. The isolation of active constituents from these hit plants is described below.

## Cardenolides from *Calotropis gigantea* (Asclepiadaceae)

**Fig. 2:**
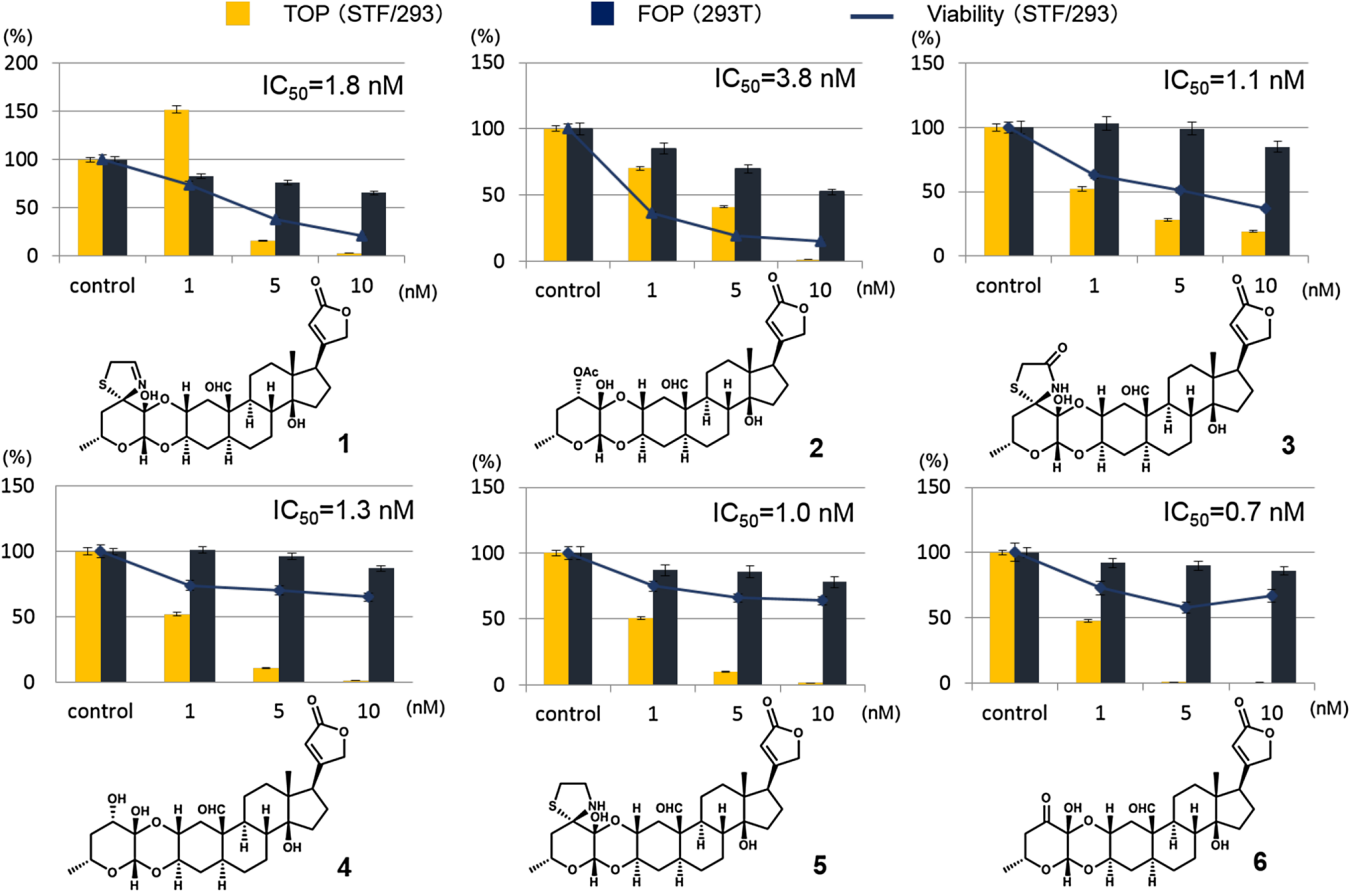
Compounds isolated from *C. gigantea* and their effect on TCF/β-catenin transcriptional activity (TOP/FOP)

A methanol (MeOH) extract of the exudate of *Calotropis gigantean* (Asclepiadaceae), which was collected in Barisal, Bangladesh, in November 2012, exhibited significant inhibition of TOP activity. After extraction with MeOH and solvent partitioning with hexane, ethyl acetate, and water, the ethyl acetate-soluble fraction proved to be active. Further fractionation of the ethyl acetate fraction using silica gel, Sephadex LH20, and ODS chromatography and guided by the same assay yielded six compounds, all of which were identified as cardenolides based on spectral data, as shown in Fig. 2 [20]. An examination of TCF/β-catenin transcriptional activity (TOP/FOP activity) of the six isolated compounds revealed that they strongly inhibited TOP activity (at nanomolar concentrations), but no substantial decrease in FOP activity was observed (Fig. 2). Compounds **4**, **5**, and **6** in particular did not significantly decrease cell viability. Therefore, these compounds were revealed to be potent TCF/β-catenin transcriptional inhibitors. All six isolated compounds exhibited cytotoxicity against three Wnt-dependent colon cancer cell lines (SW480, DLD1, and HCT116), with IC_50_ values ranging from 1.8 to 7.0 nM, but the compounds were not significantly cytotoxic to Wnt-independent RKO colon cancer cells (IC_50_ > 10 nM).

Further studies on the Wnt signaling inhibitory activity of *C. gigantea* constituents was carried out using compound **4** (calotropin) and SW480 colon cancer cells. Treatment of SW480 cells with calotropin (**4**) led to a significant dose-dependent decrease in both nuclear and cytosolic β-catenin protein expression. Calotropin (**4**) also decreased protein levels of c-myc, which is a target of Wnt, indicating that calotropin inhibits the Wnt signaling pathway. It was reported that the level of β-catenin protein is regulated by a proteasomal degradation system. The decrease in β-catenin expression mediated by calotropin (**4**) was abolished by MG-132, a proteasome inhibitor; calotropin (**4**) did not decrease β-catenin expression in the presence of MG-132. This result suggests that calotropin (**4**) accelerates the proteasomal degradation of β-catenin.

Proteasomal degradation takes place after ubiquitination of the target protein to be degraded, and ubiquitination of the protein takes place after its phosphorylation by GSK3β (glycogen synthase kinase 3β) and CK1α (casein kinase 1α). We therefore examined the level of β-catenin phosphorylation in SW480 cells as follows: (1) calotropin (**4**) induced β-catenin degradation, which was recovered using the GSK3β inhibitor LiCl; (2) calotropin (**4**) induced phosphorylation of β-catenin at the CK1α site (S45), which was not affected by LiCl; and (3) calotropin (**4**) induced phosphorylation of β-catenin at the GSK3β sites (S33, S37, and T41), which was inhibited by LiCl. It is known that β-catenin is phosphorylated first at the CK1α site, followed by phosphorylation at the GSK3β sites. These results thus indicated that calotropin (**4**) induces phosphorylation of the CK1α site of β-catenin.

The phosphorylation of β-catenin was then examined using the CK1α inhibitor CKI7, with the following results: (1) calotropin (**4**) increased the CK1α protein level, which was not affected by CKI-7; (2) calotropin (**4**) increased β-catenin phosphorylation at the CK1α and GSK3β sites, which was inhibited by CKI-7; and (3) calotropin (**4**) induced β-catenin degradation, which was recovered by CKI-7. CKI-7, a kinase inhibitor, inhibited phosphorylation by CK1α without affecting the level of CK1α protein. These results indicated that the level of CK1α protein increases as a result of treatment with calotropin (**4**).

**Fig. 3:**
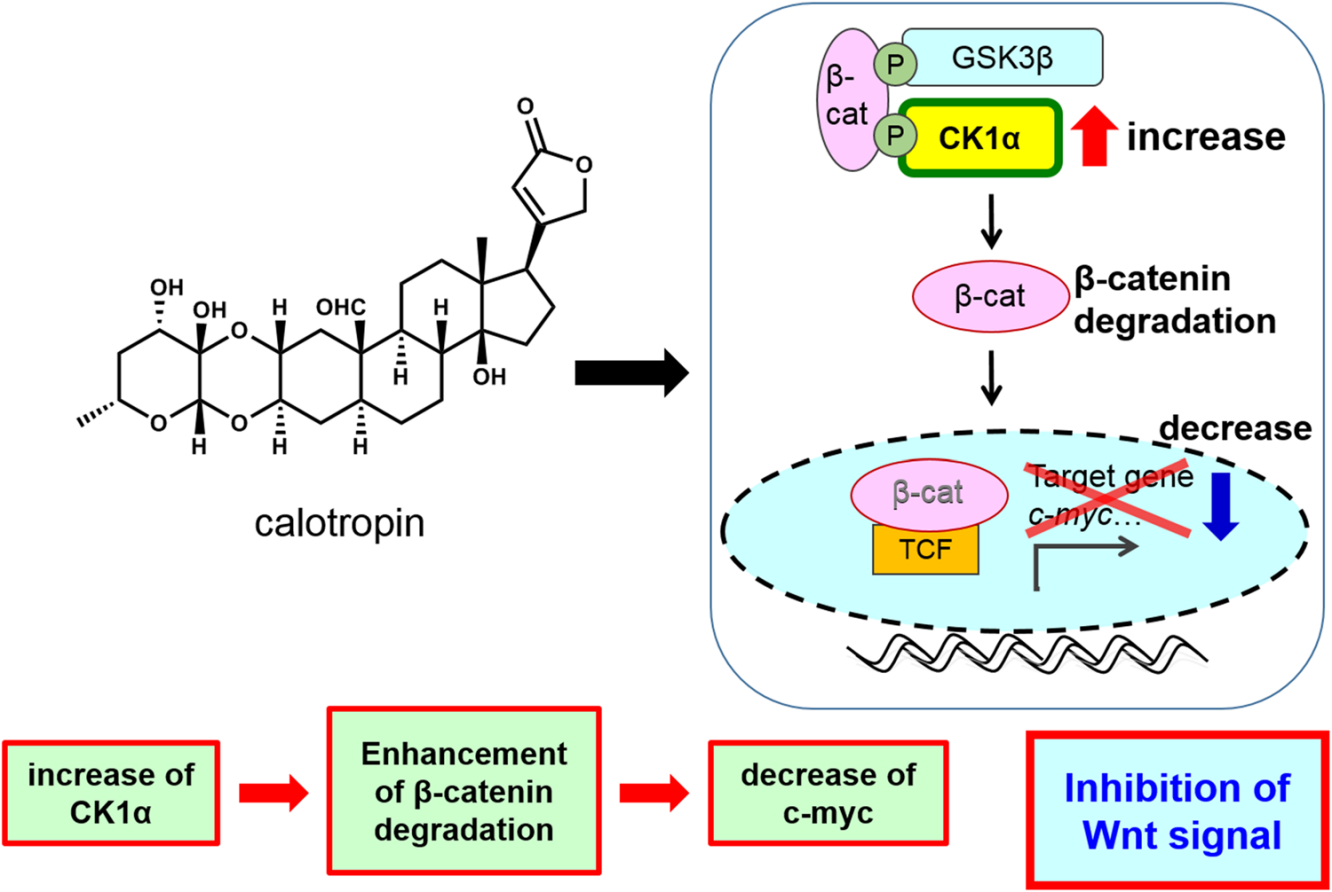
Calotropin inhibits Wnt signaling by increasing CK1α

The effect of calotropin (**4**) was further examined by knockdown of CK1α expression using siRNA. Following CK1α knockdown by siRNA, calotropin (**4**) did not induce phosphorylation of β-catenin. Subsequently, no degradation of β-catenin was observed, even in the presence of calotropin (**4**), under conditions of siRNA knockdown. These results indicated that the effect of calotropin (**4**) is dependent on CK1α. Furthermore, real-time PCR experiments revealed that calotropin (**4**) increases the expression of CK1α mRNA. From these observations, the effect of calotropin (**4**) can be explained as follows. Calotropin (**4**) induces an increase in CK1α protein levels and then induces degradation of β-catenin, followed by a decrease in the expression of genes such as c-myc. Thus, calotropin (**4**) inhibits the Wnt signaling pathway (Fig. 3).

Two other compounds that inhibit Wnt signaling via CK1α have been identified, but the mechanism of calotropin (**4**) inhibition differs from that of the other two compounds. Pyrvinium activates CK1α enzyme activity, but has no effect on CK1α protein level [21]. Honokiol increases both CK1α and GSK3β protein levels [22]. In contrast, calotropin (**4**) increases CK1α protein levels, but has no effect on GSK3β. Thus, calotropin (**4**) inhibits Wnt signaling via a different mechanism.

Flow cytometry analyses demonstrated that calotropin (**4**) also induces apoptosis of SW480 colon cancer cells. On knockdown of CK1α by siRNA, calotropin (**4**) did not induce apoptosis, thus indicating that the apoptosis-inducing effect of calotropin (**4**) is CK1α dependent.

## Diterpene from *Scoparia dulcis*

The aerial parts of *Scoparia dulcis* (Plantaginaceae) specimens collected from Khulna, Bangladesh in January 2011 were extracted with MeOH, and the extract was subjected to Diaion HP-20 column chromatography to remove chlorophyll. A fraction of this column eluted with MeOH was partitioned between hexane, ethyl acetate, 1-butanol, and water. The ethyl acetate-soluble fraction exhibited significant TOP-inhibiting activity, and this fraction was further separated using a Sephadex LH-20 column and reversed-phase HPLC to isolate the active compound, which was identified as a scopadulan diterpene, scopadulciol (**7**), based on spectral data [23].

The TOP/FOP activity of scopadulciol (**7**) was examined in AGS human gastric cancer cells, and these analyses revealed that scopadulciol (**7**) decreased TOP activity selectively, with no effect on FOP activity. Scopadulciol (**7**) also downregulated the expression of the Wnt target proteins c-myc, cyclin D1, and survivin, suggesting that scopadulciol (**7**) inhibits Wnt signaling in AGS cells. Scopadulciol (**7**) exhibited stronger cytotoxicity against Wnt-dependent AGS cells (IC_50_, 0.07 μM) than RKO cells (IC_50_, 0.48 μM), a line of Wnt-independent colon cancer cells. In AGS cells, an increase in sub-G1 populations that led to an increased rate of cell death was observed, whereas no significant change was observed in the cell cycle of RKO cells. The effects of scopadulciol (**7**) were further examined in Wnt-dependent AGS cells. Western blot analyses using the whole-cell lysate showed that scopadulciol (**7**) decreases the β-catenin level and also inhibits the nuclear accumulation of β-catenin. We then examined whether the decrease in β-catenin levels mediated by scopadulciol (**7**) involves proteasomal degradation using the proteasome inhibitors MG132 and epoxomicin. These proteasome inhibitors abrogated the decrease in β-catenin level mediated by scopadulciol (**7**), suggesting that enhanced β-catenin degradation induced by scopadulciol (**7**) is proteasome dependent.

The above experiments were carried out in Wnt-dependent AGS cells, but AGS cells are also known to exhibit strong resistance to TRAIL, a death ligand involved in signaling mechanisms associated with cancer-selective apoptosis [24]. The use of TRAIL is considered an attractive therapeutic strategy, but some cancer cells, such as AGS, are known to be TRAIL resistant. We therefore examined the effect of scopadulciol (**7**) on TRAIL resistance. The combination of scopadulciol (**7**) with TRAIL resulted in significantly enhanced cytotoxicity against AGS cells, indicating that scopadulciol (**7**) suppresses the TRAIL resistance of AGS cells. In addition, flow cytometry analyses revealed that treatment of AGS cells with scopadulciol (**7**) in the presence of TRAIL resulted in an increase in apoptosis. In the presence of the caspase inhibitor z-VAD-fmk, the cytotoxicity of compound **7** in combination with TRAIL was reduced, thus indicating that apoptosis induced by combined use of scopadulciol (**7**) and TRAIL is mediated by caspase.

**Fig. 4:**
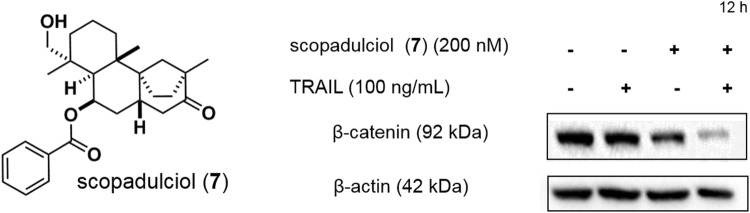
Combined treatment with scopadulciol (**7**) and TRAIL enhances β-catenin degradation in AGS cells

Experiments examining the effect of scopadulciol (**7**) on apoptosis-related protein levels revealed that treatment of AGS cells with scopadulciol (**7**) increased the levels of DR4 and DR5 (death receptors of TRAIL) and decreased the level of the anti-apoptotic protein Bcl2. These observations were consistent with data showing that scopadulciol (**7**) enhances TRAIL-induced apoptosis. In addition, combined treatment of AGS cells with scopadulciol (**7**) and TRAIL resulted in enhanced β-catenin degradation (Fig. 4). The relationship between Wnt signaling and TRAIL has not been fully elucidated, but it is possible that the inhibition of Wnt signaling contributes to TRAIL-induced apoptosis.

## Lymonoids from *Xylocarpus granatum* and *Azadirachta excelsa*

**Fig. 5:**
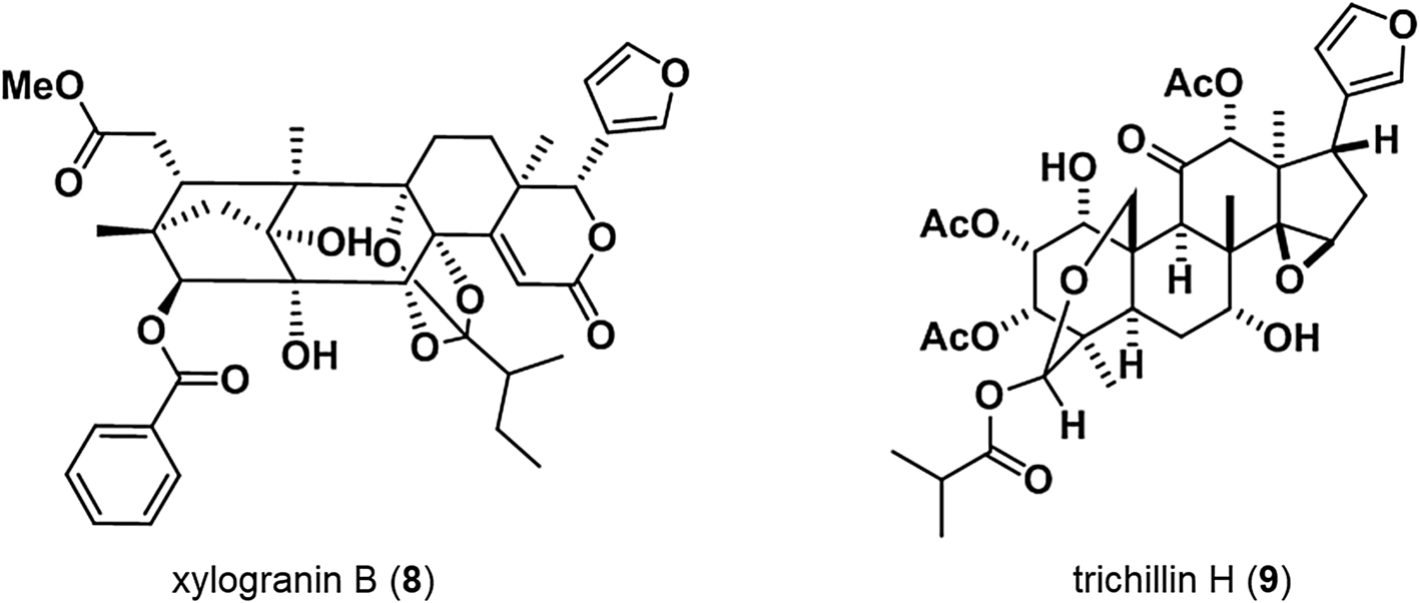
Xylogranin B and trichillin H

Activity-guided fractionation of MeOH leaf extracts of *Xylocarpus granatum* (Meliaceae) specimens collected from the Sundarbans Mangrove Forest, Bangladesh, in November 2008 led to the isolation of four limonoids, including two newly discovered compounds, which were designated xylogranin A and B [25]. Xylogranin B (**8**) (Fig. 5) inhibited TOP activity, with an IC_50_ of 48.9 nM, and exhibited strong cytotoxicity against SW480 colon cancer cells. Xylogranin B (**8**) possesses a benzoyl ester group at C-3 position instead of a tigloyl group of swietephragmin C, which was isolated together with compound **8** from *X. granatum* [25]. Compound (**8**) significantly decreased β-catenin protein levels in the nucleus (but not the cytosol) of SW480 cells, indicating that the Wnt signaling inhibitory effect of xylogranin B (**8**) involves decreased β-catenin levels in the nucleus. Treatment with xylogranin B (**8**) led to a significant decrease in levels of c-myc and PPARδ, the products of several genes targeted by Wnt, and treatment with xylogranin B (**8**) suppressed *c*-*myc* and *PPARδ* mRNA expression.

Trichillin H (**9**) (Fig. 5) [26] was isolated from the dried fruit of *Azadirachta excels* (Meliaceae) specimens collected in Khulna, Bangladesh, in February 2012. Trichillin H (**9**) inhibited TOP activity, with an IC_50_ of 0.3 μM, but did not significantly affect FOP activity. Compound (**9**) exhibited strong cytotoxicity against Wnt-dependent AGS and HCT116 cells (IC_50_ of 0.24 and 0.16 μM, respectively) and weak cytotoxicity against Wnt-independent RKO cells (IC_50_, 0.4 μM). Trichillin H (**9**) did not affect the level or localization of β-catenin, but downregulated the expression of c-myc (a target of Wnt) in HCT116 colon cancer cells, suggesting that trichillin H (**9**) inhibits Wnt signaling by affecting components downstream of β-catenin.

## Alkaloids from *Eurycoma longifolia* and *Tabernaemontana divaricata*

**Fig. 6:**
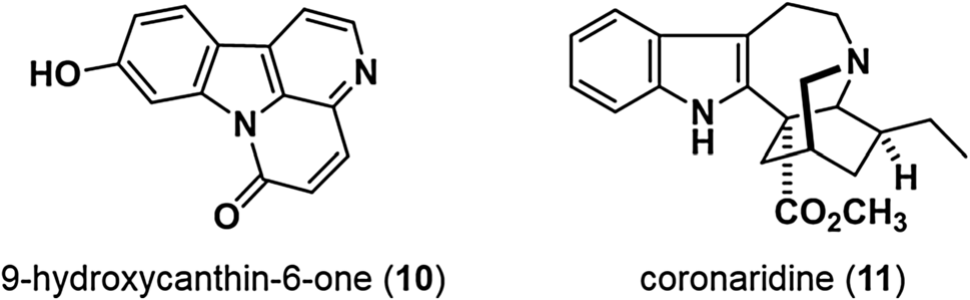
9-Hydroxycanthin-6-one and coronaridine

The MeOH extract of air-dried roots of *Eurycoma longifolia* (Simaroubaceae), collected from Khon Kaen, Thailand, was partitioned with hexane, ethyl acetate, and 1-butanol. Activity-guided fractionation of the hexane and ethyl acetate layers using silica gel and ODS column chromatography, followed by preparative HPLC, led to the isolation of a compound that inhibited TOP activity with an IC_50_ of 6.3 μM. This active compound was identified as a β-carboline alkaloid, 9-hydroxycanthin-6-one (**10**) (Fig. 6), based on spectral data [27]. Treatment of SW480 colon cancer cells with 9-hydroxycanthin-6-one (**10**) led to a significant decrease in the level of β-catenin protein and increased levels of phosphorylated β-catenin (p-β-catenin) (Ser33, Ser37, Thr41), without any effect on levels of GSK3β, CK1α, and p-β-catenin (Ser45). Intracellular β-catenin levels are regulated by the ubiquitin–proteasome system. β-Catenin is first phosphorylated on Ser45 by CK1α, followed by phosphorylation on Thr 41, Ser37, and Ser33 by GSK3β. Phosphorylation of these residues is essential for the recognition of β-catenin by the F-box β-transducin repeat-containing protein (β-TrCP), leading to ubiquitination and degradation by the proteasome pathway.

The degradation of β-catenin by 9-hydroxycanthin-6-one (**10**) was suppressed by GSK3β-siRNA but not CK1α-siRNA. These results suggest that 9-hydroxycanthin-6-one (**10**) inhibits Wnt signaling via activation of GSK3β enzyme activity independent of CK1α. The effect of 9-hydroxycanthin-6-one (**10**) on zebrafish was also investigated. 9-Hydroxycanthin-6-one (**10**) decreased the expression of the Wnt target genes *mitf* and *zic2a* in zebrafish embryos and induced malformation of the MHB (midbrain-hindbrain boundary) and formation of a curled tail in zebrafish embryos. 9-Hydroxycanthin-6-one (**10**) also decreased TOPdGFP activity in TOPdGFP-transgenic zebrafish embryos. Although treatment of zebrafish embryos with BIO (6-bromoindirubin-3′-oxime, an inhibitor of GSK3β) alone induced an eyeless phenotype, additional treatment with 9-hydroxycanthin-6-one (**10**) rescued the eyeless phenotype caused by BIO. These observations indicate that 9-hydroxycanthin-6-one (**10**) inhibits Wnt signaling in vivo.

Coronaridine (**11**) (Fig. 6) [28] was isolated from the MeOH extract of the air-dried aerial parts of *Tabernaemontana divaricata* (Apocynaceae), collected in Bangladesh in 2011. Coronaridine (**11**) inhibited TOP activity with an IC_50_ of 5.8 μM. Coronaridine (**11**) also decreased β-catenin protein levels in SW480 colon cancer cells, and this decrease was not affected by co-treatment with MG132 (a proteasome inhibitor). In addition, treatment of SW480 cells with coronaridine (**11**) caused a decrease in β-catenin mRNA expression. These results suggest that coronaridine (**11**) inhibits the Wnt signaling pathway by decreasing the expression of β-catenin mRNA.

## Lignan from *Hibiscus ficulneus*

**Fig. 7:**
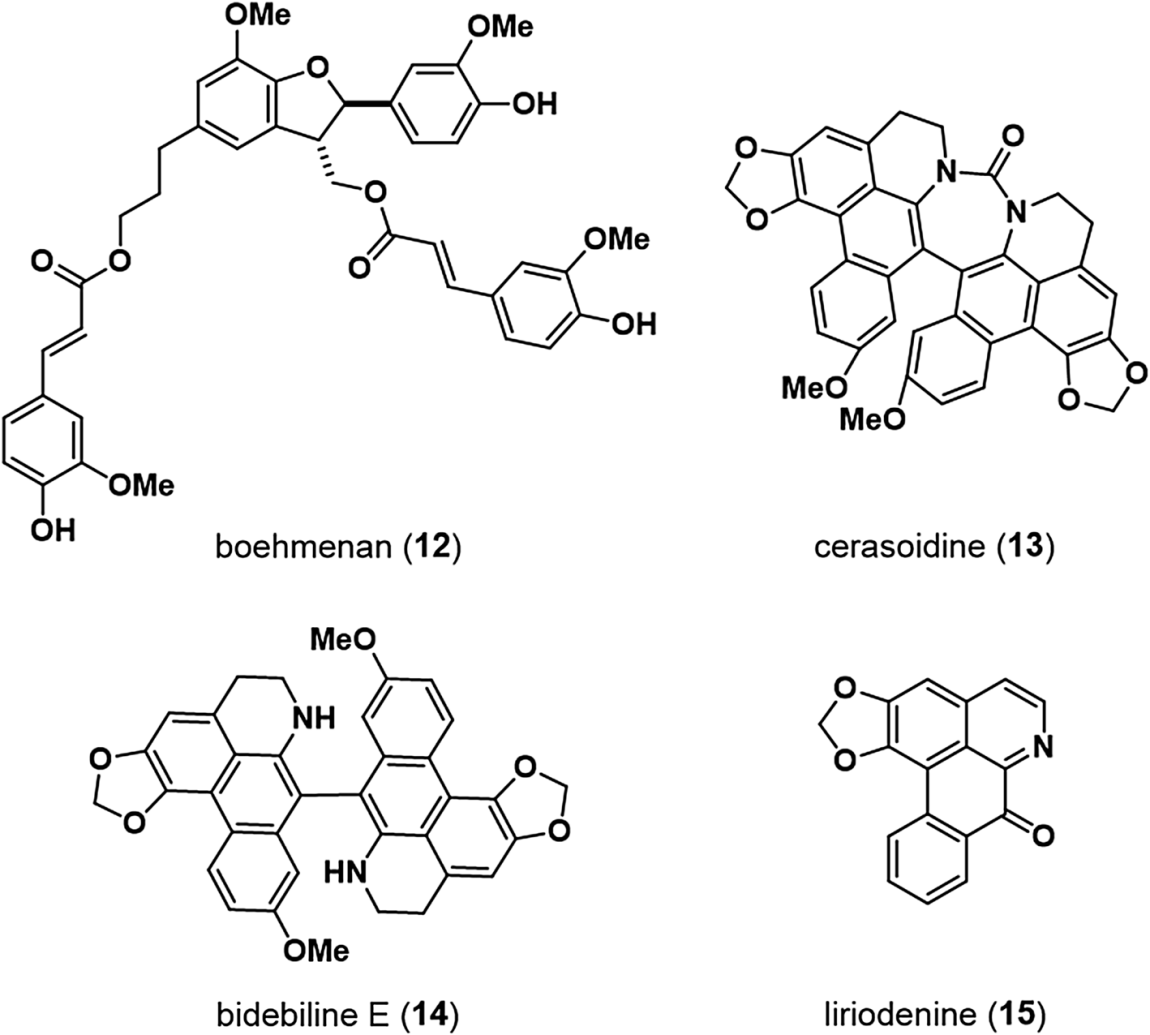
Boehmenan and aporphine alkaloids

Activity-guided separation based on TOP-inhibition activity in the MeOH extract of stems of *Hibiscus ficulneus* (Malvaceae) specimens collected in Bangladesh in 2010 led to the isolation of four lignans exhibiting dose-dependent TOP-inhibition activity without significant effects on FOP activity. Of these four lignans, boehmenan (**12**) (Fig. 7) [29] exhibited an IC_50_ of 1.0 μM against TOP activity and cytotoxicity against both Wnt-dependent HCT116 colon cancer cells and Wnt-independent RKO colon cancer cells, with IC_50_ values of 22.8 and 25.4 μM, respectively. Western blot analyses showed that boehmenan (**12**) decreased the levels of both cytosolic and nuclear β-catenin in STF/293 cells and decreased levels of c-myc protein, a TCF/β-catenin target. In addition, the decrease in β-catenin levels mediated by boehmenan (**12**) was abolished by co-treatment with the proteasome inhibitor MG132. Thus, the downregulation of β-catenin levels by boehmenan (**12**) may be related to increased proteasomal degradation.

## Aporphine alkaloids from *Polyalthia cerasoides*

MeOH and CHCl_3_ extracts of dried roots of *Polyalthia cerasoides* (Annonaceae) specimens collected in the Khon Kaen area of Thailand exhibited significant TOP-inhibition activity. Activity-guided fractionation using an ODS column and preparative HPLC resulted in the isolation of a new as well as a previously described bis-aporphine alkaloid (**13** and **14**, respectively). The structure of the new compound, which was designated cerasoidine (**13**), was determined by X-ray analysis as a novel dimeric structure with a unique ureido bond. Cerasoidine (**13**) was revealed as a 57:43 enantiomeric mixture by chiral HPLC analysis. The known bis-aporphine alkaloid, bidebiline E (**14**), markedly inhibited Wnt signaling by suppressing the accumulation of β-catenin in the nucleus. Liriodenine (**15**), an aporphine alkaloid with a monomeric structure, was also obtained from *P. cerasoides*. Studies suggested that liriodenine (**15**) inhibits Wnt signaling via up-regulation of the proteasome system to increase β-catenin degradation [30].

## Ricinine from *Ricinus communis* (Wnt activator)

**Fig. 8:**
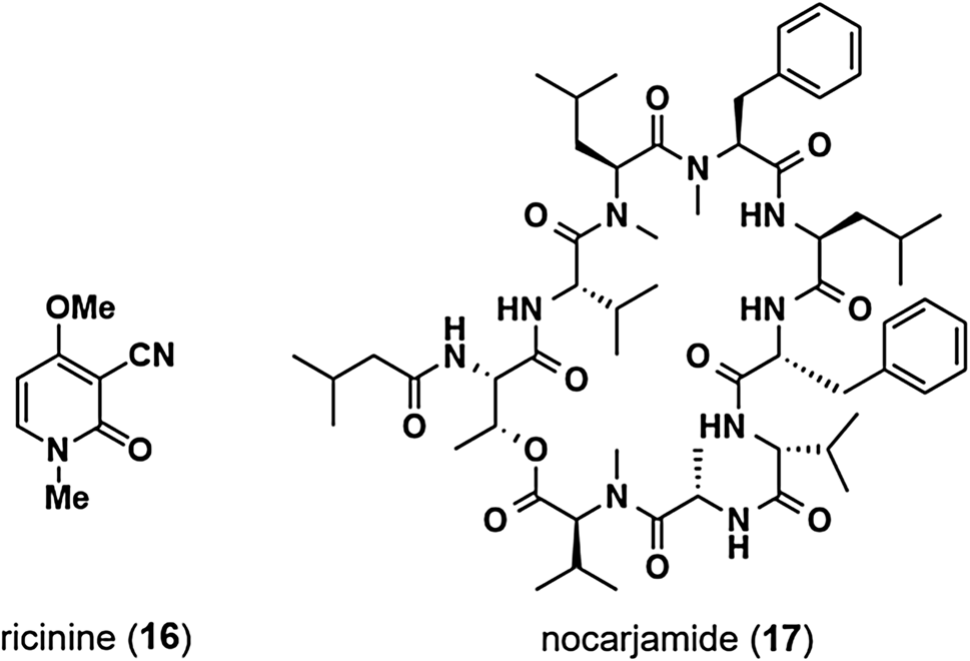
Ricinine and nocarjamide

Aberrant activation of Wnt signaling is related to the development of tumors and other diseases, whereas aberrant downregulation of Wnt signaling is also associated with various diseases, including osteoporosis, tetra-amelia, and Alzheimer's disease. Wnt signaling activators are therefore sought as potentially attractive therapeutic agents and useful tools for biological research. In our screening for effects on TOP activity, the MeOH extract of the stems of *Ricinus communis* (Euphorbiaceae) specimens collected in Bangladesh in 2010 was found to increase TOP activity twofold at 100 μg/mL. Activity-guided fractionation of the EtOAc-soluble fraction of the extract, which induced a 2.2-fold increase in TOP activity at 25 μg/mL, using silica gel, ODS column chromatography, and reversed-phase preparative HPLC led to the isolation of four compounds, including ricinine (**16**) (Fig. 8) [31] and three triterpenoids. Ricinine (**16**) enhanced TOP activity in a dose-dependent manner, with no effect on FOP activity. Western blot experiments revealed that ricinine (**16**) increased the β-catenin protein level and decreased levels of p-β-catenin phosphorylated on Ser33, Ser37, Thr41, and Ser45, whereas protein levels of GSK3β and CK1α were not affected by ricinine (**16**). The effect of ricinine (**16**) in the presence of a CK1α activator, pyrvinium, was examined next. Treatment with ricinine (**16**) in the presence of pyrvinium resulted in greater decreases in the levels of p-β-catenin phosphorylated on Ser33, Ser37, Thre41, and Ser45 compared with treatment with pyrvinium alone. In contrast, β-catenin levels on combined treatment with ricinine (**16**) and pyrvinium were higher than those observed following treatment with pyrvinium alone. These data suggest that ricinine (**16**) activates Wnt signaling via inhibition of CK1α enzyme activity. Our analyses also revealed that ricinine (**16**) increases β-catenin protein levels in zebrafish embryos in a dose-dependent manner.

## Nocarjamide from *Nocardia tenerifensis*

We recently initiated a study of pathogenic actinomycetes *Nocardia* sp. as unutilized resources for the isolation of bioactive natural products [32, 33] and developed a co-culture method for *Nocardia* sp. with animal cells to isolate compounds that are not produced in *Nocardia* sp. in mono-culture [34]. Co-culture of *Nocardia tenerifensis* IFM 10554^T^ with mouse macrophage-like J774.1 cells in modified Czapek-Dox medium resulted in the isolation of a new cyclic nonapeptide, nocarjamide (**17**) (Fig. 8), the structure of which was firmly established by X-ray analysis [35]. Nocarjamide (**17**) possesses nine amino acid residues connected by eight amide bonds, while the *N*-terminal threonine residue was connected with 3-methylbutanoic acid moiety and the C-terminal *N*-methylvaline residue with an ester bond. Of nine amino acids, two residues (phenylalanine and one of two valine residues) prove to be D. In addition, the X-ray analysis of nocarjamide (**17**) revealed that two conformers are present in a 1:1 ratio in its crystal state. Although nocarjamide (**17**) exhibited almost no cytotoxicity against several cancer cell lines, TOP activity in the presence of 20 and 40 μM nocarjamide (**17**) was 1.8- and 2.2-fold higher than that of the control, respectively. At both of these concentrations, the TOP activity was 1.5-fold higher than the FOP activity. Western blot analyses of HEK293 cells showed that nocarjamide (**17**) increased protein levels of β-catenin and c-myc, suggesting that nocarjamide (**17**) activates Wnt signaling.

## Conclusion

Screening studies of extracts of various medicinal plants and actinomycetes using a cell-based luciferase assay assessing TCF/β-catenin transcriptional activity (TOP activity) resulted in the isolation of a number of bioactive constituents that affect the Wnt signaling pathway. These active constituents affect Wnt signaling via different mechanisms, including increasing CK1α protein (calotropin) levels, activating GSK3β enzyme activity (9-hydroxycantin-6-one), decreasing nuclear β-catenin levels (xylogranin B), decreasing β-catenin mRNA expression (coronaridine), and enhancing β-catenin degradation (scopadulciol, boehmenan, and liriodenine). Our screening also identified a Wnt signaling activator that functions via inhibition of CK1α activity.

Further natural products-based screening studies are in progress aimed at discovering compounds exhibiting unique effects on various signaling pathways that are expected to be useful in basic biological research and disease therapy.
